# Health-Related Quality of Life in Children with Overweight and Obesity: An Explorative Study Focused on School Functioning and Well-being

**DOI:** 10.5334/cie.58

**Published:** 2023-07-14

**Authors:** Livia Buratta, Elisa Delvecchio, Michele Capurso, Claudia Mazzeschi

**Affiliations:** 1 University of Perugia, IT

**Keywords:** childhood obesity, health-related quality of life, school functioning, school well-being, self-esteem, social relationships

## Abstract

Overweight and obesity in childhood has reached epidemic levels, and their roles in physical and psychological health are now recognized. Recently, researchers have focused on the impact of these weight problems in health-related quality of life (HRQoL) domains, which are less investigated in children. This exploratory study examined the differences in HRQoL domains between a clinical group who were overweight/obesity treatment-seeking (n = 58) and a normal-weight group (n = 44) in a sample of 102 children, with a specific focus on school functioning and well-being. The second aim explored the link between our findings and other HRQoL dimensions. After controlling for sex and age, a multivariate analysis of variance showed lower levels in school functioning and well-being dimensions between overweight/obesity than normal-weight (F = 4.72; p < 0.05). Correlation analyses highlighted positive links between lower school functioning and well-being and lower levels of self-esteem (r = 0.308; p < 0.01) and social domains in terms of friendships (r = 0.522 ; p < 0.001) and family relationships (r = 0.561; p < 0.001) in children who were with overweight and obesity. This study discusses the implications of these findings in educational research and practices.

## Introduction

Over the last two decades, pediatric cases of being overweight and obese have reached epidemic levels, particularly in more developed countries. According to age and sex based on the World Health Organization, child growth references considered normal weight children with a BMI Zscore from –1 to +1, and overweight/obesity, children with a BMI Zscore >1. In European countries, one in three children is overweight or obese, and future predictions are not encouraging. Some estimates suggest that 254 million children and adolescents aged 5–19 will suffer from obesity by 2030 (Lobstein & Brinsden, 2019). It is now established that pediatrics being with overweight and obesity are risk factors for several non-communicable diseases and early death in adulthood. For these reasons, it is considered one of the most severe health problems of the 21^st^ century. Excess weight is related to physical issues and has significant psychosocial consequences (Puhl & Latner, 2015). Furthermore, a review by Buttitta, Iliescu, Rousseau & Guerrien (2014) showed that being with overweight and obesity in childhood also impacted the HRQoL.

The HRQoL is an essential measure of people’s health and an indicator of the global burden of health problems over time (Buttitta et al., 2014). The HRQoL is a multidimensional construct consisting of physical, emotional, social well-being, and daily functioning, perceived by people. It comprises how people feel psychologically and physically, how they feel in relation to other people, and cope with age-related everyday life situations (Ravens-Sieberer et al., 2007). A deeper exploration of HRQoL in childhood, includes the core domains and aspects characterizing this specific life period. These domains include physical, emotional, and social well-being, alongside self-perception and school functioning/well-being. The latter considers the individual evaluations of cognitive and affective experiences in school or linked to school (Raven-Sieberer et al., 2007; Obermeier & Glaser-Zikuda, 2022). Studies using the HRQoL in pediatric populations are very recent, and the evidence remains unclear.

In their review, Buttitta and colleagues (2014) identified the domains most affected by body weight issues in the HRQoL. The studies analyzed in this review showed that children and adolescents who were with overweight and with obesity reported lower scores in the self-perception, emotional and social domains. The social domains mainly refer to family life and relationships with peers. However, the school functioning data showed mixed results. This dimension appears more fragile in a clinical population with obesity that is also treatment-seeking. They experienced more cognitive, learning, and concentration difficulties and anxiety at school than their healthy-weight peers. Contrarily, a recent paper (Zanganeh et al., 2022) did not report any effects of being with overweight and obesity on the HRQoL dimensions, including school functioning and well-being. As suggested by the authors, environment and culture may play crucial roles in these differences. Several studies highlight that culture can influence the perception of being with overweight and obesity and their different risk aspects (Caprio et al., 2008). In some cultures, childhood obesity is not perceived as unhealthy; instead, they believe it to be a sign of good health (MacArthur et al., 2004; Pallan et al., 2012). These cultural differences may result in different links between the HRQoL and obesity.

To our knowledge, few studies have explored the link between weight status and the HRQoL dimensions in Italian children, all were conducted on school-based populations (Mastorci et al., 2021; Masini et al., 2021). These studies did not show any differences between children and adolescents with overweight and obesity compared to their normal-weight peers for school functioning and well-being. A possible explanation is that the community-based study recruits treatment-seeking children and adolescents and those who do not seek clinical help. Children and adolescents with overweight and obesity who seek treatment may have a worse HRQoL than those who do not seek treatment (Buttitta et al., 2014). These results suggest the need for further studies explicitly focused on clinical populations, preferably using self-report measures as in the studies mentioned above (Buttitta et al., 2014). Indeed, the authors highlighted that parents tended to underestimate their children’s HRQoL, while the children tended to report higher HRQoLs than their parents. As suggested by Raven-Sieberer and colleagues (2007), these results endorse the importance of listening to children as informants for a broader understanding of the quality of life from their perspective. This self-informed reporting may be more critical in a clinical setting where the parents’ perspectives could be influenced by personal perceptions and experiences of their children’s health status (Helseth, Haraldstad & Christophersen, 2015).

### The Present Study

This study’s first aim was to investigate differences in the HRQoL dimensions in a sample of Italian children aged 6–14 years. This was assessed by self-reporting between subjects with overweight and obesity, recruited from a clinical center and those of a normal weight recruited from the general population and considering the effects of sex and age. We expected that children with overweight/obesity would score lower levels of self-esteem, emotional well-being, social domains, and school functioning due to being from a clinical population. Moreover, in line with the previous literature, we hypothesized lower levels in girls than boys, particularly for self-esteem and social domains (Buttitta et al., 2014).

This study’s second aim was to explore the relationship between school functioning and well-being, and all the other HRQoL dimensions. Correlation analyses were conducted separately in normal-weight and overweight/obesity groups. Considering the interplay of individual and context/social variables on scholastic well-being (Hascher, Morinaj-Turkin & Waber, 2018), we assumed there would be links between school functioning and self-perception, the family and peers’ environment, and relationships.

## Method

### Participants

**Figure 1:**
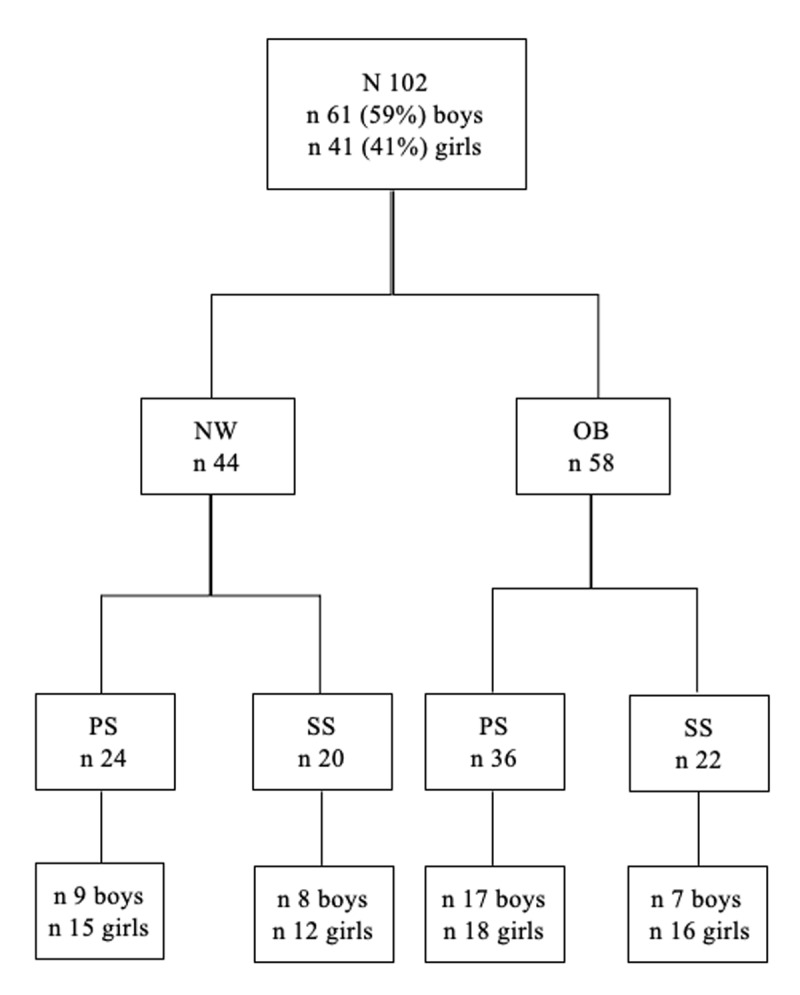
Sample distribution flow chart. *Note*: NW = Normal weight; OB = Overweight/Obesity; PS = Primary School; SS = Secondary School.

The sample of this cross-sectional study was composed of 102 children, Figure 1 can help to better identify the distribution of the sample. 61 boys (59%) and 41 girls (41%), with a mean age of 10.3 (Sd 1.89; Min 6 – Max 14). Fifty-eight children with overweight or obesity (OB) with a mean BMI Zscore of 1.82 (Sd = .82) and forty-four were with normal weight (NW) with a mean BMI Zscore of 0.22 (Sd = .60). The chi-square showed no differences in the distribution between clinical and healthy group (χ^2^ = 1.92; *p* = .166). The two groups have been balanced for sex (χ^2^ = 0.08; *p* = .780) and age (t = 0.18; *p* = .853). Fifty-eight children attended primary school, from 6 to 10 years old (PS), and forty-for the secondary school, from 11 to 14 years old (SS); the chi-square showed no differences in the distribution between PS and SS (χ^2^ = 1.92; *p* = .166).

### Procedure

Children with overweight or obesity were recruited at the Healthy Lifestyle Institute of the University of Perugia (C.U.R.I.A.Mo. – Centro Universitario Ricerca Interdipartimentale Attività Motoria). In contrast, children with normal weight were recruited from two pediatric clinics. Data has been collected after obtaining parental informant consent. No incentives were given, and it was emphasized that participation in the study was voluntary and that children could withdraw at any moment. The study was conducted in compliance with the ethical standards for research outlined in the Ethical Principles of Psychologists and Code of Conduct of the American Psychological Association.

### Measures

**Anthropometric Measures:** Children’s height and body weight were assessed by physicians of the C.U.R.I.A.Mo. for children with obesity, and pediatricians for children with normal weight. Both cases used the standard method to calculate BMI and BMI Zscore to classify children’s BMI into normal-weight and obesity categories, according to the WHO classifications considering gender and age (De Onis, Onyango, Borghi, Siyam, Nishida & Siekmann, 2007).

**Self-report Questionnaire:** Children filled in a brief booklet composed of a schedule to collect demographic information (age and sex) and a self-report to measure the dimensions of the child’s quality of life (HRQoL), the ***Kid-Kindl***. Kid-Kindl was composed of 24 items that assess, using a 5-point Likert scale from 1 (never) to 5 (all the time), six different dimensions of the QoL: physical well-being -PW (e.g.: I felt strong and full of energy), emotional well-being – EW (e.g.: I had fun and laughed a lot), self-esteem- SE (e.g.: I felt pleased with myself), family life – FA (e.g.: I felt fine at home), friend relationship -FR (e.g.: I got along well with my friends) and school functioning and well-being – SC (e.g.: I enjoyed my lessons). All scales are added together to generate a Total Score. High values (range 0–100), indicate a good HRQoL. This questionnaire showed good internal consistency (all subscales > 0.60 and total score > 0.80). The Italian translation is available on the official website (Ravens-Sieberer & Bullinger, 1998).

### Data Analysis

Initially, a Multivariate Analysis of Variance (MANOVA) with three fixed factors was run to explore the differences in the QoL’ domains assessed with the Kid-Kindl, between obesity and normal-weight group (NW vs OB), boys and girls (B vs G) and finally between primary and secondary school children (PS vs SS). Moreover, MANOVA investigated the interactions among the fixed factors. Subsequently, Spearman’s rank correlation analysis was conducted separately for groups NW and OB, to explore the relationships between school functioning and well-being with all other HRQoL dimensions. Only the correlations with medium and strong effect sizes will be considered, in line with Cohen (1988), where 0.10, 0.30, and 0.50 represented small, medium, and strong effects sizes, respectively. All analyses were carried out with Statistical Package for Social Science version 26, IBM, NY, USA.

### Results

The MANOVA showed some differences (see Table 1). In particular, about group factor (NW vs OB), children with obesity referred to lower levels only in school well-being (Mn = 64.1, Sd = 20.4) than normal-weight children (Mn = 73.7, Sd = 15). Differences between sexes: boys had higher self-esteem (Mn = 71.5, Sd = 21.1) and higher well-being in friendship (Mn = 78.8, Sd = 13.4), than girls (SE: Mn = 58.3, Sd = 26,5; FR: Mn = 71,7, Dd = 16,6). There have been no differences between primary and secondary school children and no interactions between three fixed factors.

**Table 1:** Multivariate Analysis of Variance (MANOVA) of the QoL dimensions with three fixed factors.


KID-KINDL DIMENSIONS	TOTAL SAMPLE	GROUP NW VS OB		SEX B VS G		AGE PS VS SS	

	Mn ± Sd	F	*p*	F	*p*	F	*p*

PW	72.4 ± 22.5	0.04	0.84	0.01	0.92	0.06	0.80

EW	76.1 ± 17.2	2.40	0.12	3.62	0.06	0.01	0.93

SE	63.5 ± 25.3	1.48	0.23	8.37	0.005	1.81	0.18

FA	74.7 ± 16.3	0.36	0.55	0.50	0.48	0.04	0.83

FR	74.6 ± 15.7	0.69	0.41	4.27	0.04	0.05	0.83

SC	68.3 ± 18.8	4.72	0.03	0.20	0.66	1.99	0.16


*Note*: NW = Normal weight; OB = Overweight/Obesity; B = Boy; G = Girl; PS = Primary School; SS = Secondary School; PW = Physical Well-being; EW = Emotional Well-Being; SE = Self-esteem; FA = Family Relationship; FR = Friends Relationship; SC = School Functioning and Well-being.

Spearman’s rank correlation coefficients were calculated between SC and all HRQoL dimensions separately for NW and OB (see Table 2). Some correlations have been found only in children with overweight and/or obesity. The SC dimension positively correlates with an effect size from moderate to strong with the other three HRQoL dimensions. Lower levels in school functioning and well-being are accompanied by lower scores in self-perception and lower scores in two dimensions of social domains, family life, and friend relationships. No correlations have emerged in the normal-weight group.

**Table 2:** Spearman’s rank correlation coefficient, between School and all other HRQoL dimensions in normal-weight group and overweight/obesity group separately.


NW		PW	EW	SE	FA	FR

SC	Spearman’s rho	0.021	–0.171	0.083	0.256	0.276

**OB**		**PW**	**EW**	**SE**	**FA**	**FR**

SC	Spearman’s rho	0.278	0.262	0.308*	0.561**	0.522**


*Note*: NW = Normal weight; OB = Overweight/Obesity; PW = Physical Well-being; EW = Emotional Well-Being; SE = Self-esteem; FA = Family Relationship; FR = Friends Relationship; SC = School Functioning and Well-being. * *p < .01*: ** *p < .001*:

## Discussion

This study investigated the link between body weight and HRQoL self-reported by Italian children in a clinical population who were with overweight and obesity, focusing on the specific dimension of school functioning and well-being. Second, we analyzed the link between school functioning and well-being with other HRQoL dimensions to explore their possible simultaneous involvement in pediatric obesity and being overweight.

Contrary to expectations, the main finding showed how children who were with overweight or obesity self-reported lower levels only of school functioning and well-being than their healthy-weight peers, with no effects of sex and age. Being with overweight and obesity did not appear to affect the other HRQoL dimensions. These data agreed with previous research conducted in a clinical population that highlighted more perceived difficulties and anxiety at school (Buttitta et al., 2014). However, our results are in contrast with studies that stated that children from clinical centers reported poorer quality of life in all HRQoL dimensions (Riazi, Shakoor, Dundas, Eiser & McKenzie, 2010) and all other dimensions aside from school functioning (Boyle, Jones & Walters, 2010). Furthermore, our results were also contrary to other studies conducted in the Italian context (Mastorci et al., 2021), which highlighted differences in the association between HRQoL and body weight status, particularly regarding psychological variables. However, there were no differences for school dimensions, most likely because the previous study’s sample was recruited from a community population. In contrast, school functioning appears more impaired in a clinical population. As suggested by Zanganeh and colleagues (2022), the relationship between weight status and HRQoL may be affected by sex, age, and contextual factors related to the environment, culture, and setting.

This study’s findings regarding the effects of sex and age on HRQoL were in line with most other studies focused on quality of life (Buttitta et al., 2014). Their results generally showed higher self-esteem and social relationship dimensions in boys than girls. Concerning school functioning, boys and girls reported similar perceptions regardless of body weight status. As in a previous study, we found no differences between children from primary and secondary school; however, there were differences between children aged under 15 years and adolescents over 15 years (Keating, Moodie & Swinburn, 2011).

Children’s subjective perception of school functioning and well-being depends on two main factors: cognitive, including a self-concept of academic achievement and positive attitudes towards school, worries about school, and affective factors that focus on enjoyment in school (Obermeier & Zikuda, 2022). Being with overweight and obesity correlate with more severe difficulties in academic performance, and children with weight problems showed lower scores on math exercises than normal-weight children. Children with overweight and obesity are more likely to develop a poor working memory, possibly closely related to adverse academic outcomes (Wu, Chen, Yang & Li, 2017) and poorer perceptions of school functioning and well-being.

Besides dissatisfaction in children with obesity on their academic performance, school functioning and well-being there may also be related effects on individual aspects, like self-concept and interpersonal aspects, such as social relationships, both in school (with classmates and teachers) and outside of school (with family) (Hascher et al., 2018). Low self-esteem and social difficulties are often present in children with overweight and obesity (Griffiths, Parsons & Hill, 2010). Our results showed no differences in self-esteem and social domains between children with overweight and obesity compared to normal-weight children. However, as expected, the analysis between the school dimension and other HRQoL domains highlighted three correlations. A further reduction in school functioning and well-being scores was also associated with lower self-esteem and social dimensions regarding relationships with friends and family. Children with overweight and obesity experience more difficulties completing their schoolwork; they worry about bad marks, show little enjoyment in school, and find it more difficult to feel proud of themselves. Furthermore, they may experience more difficulty getting along well with their friends and generally feel different. Moreover, children who functioned less well in school and had poorer well-being often came from homes with a poor family climate, characterized in the *Kid-Kindl* by multiple conflicts and more parental restrictive behaviors. These results can be interpreted in relation to the western sociocultural context in which the thinness model dominates increasing the risk of social rejection for children and adolescents with overweight and obesity, who are often teased for their weight status by peers and family members (Eisenberg et al., 2003). This compromises their relationships with their parents and peers and may decrease their self-esteem, negatively impacting the HRQoL and their functioning, particularly in their main life context of school (Puhl and Suh, 2015).

A recent study on chronic disease in children emphasized that higher school functioning and well-being and a higher perception of a favorable family context, in terms of positive relationships, are essential to improve children’s conceptions of themselves. In turn, children with higher self-esteem showed positive life outcomes such as academic achievements and satisfaction in their relationships with others (Tremolada, Taverna, Bonichini, Basso & Pillon, 2017). Being with overweight and obesity are considered chronic diseases (Burki, 2021). Several researchers stressed that children with excess weight reported HRQoL levels comparable to children with other chronic diseases, such as cancer (Schwimmer, Burkwinkle & Varni, 2003). Therefore, we hypothesized the same interplays among individual factors, relationships with others, scholastic and family well-being. The present cross-sectional exploratory study lays the foundations for future research to deepen our understanding of school well-being, its components, and the complex network of related factors. It remains rarely studied in general and particularly in the context of childhood obesity.

### Limits of the Study

This study has some limitations. First, the study is exploratory, and the findings must be replicated with a larger clinical sample of children with overweight and obesity to confirm any findings. Second, the generalizability of the results is limited. Thirdly, only the perspectives of children and adolescents were considered, and only self-report measures used.

Future studies should incorporate the perspectives of parents and teachers as informants to give a complete picture of the school’s well-being dimension. Future studies should also consider socio-demographic and cultural factors that might shape children’s perceptions of their HRQoL. Evaluation should include an assessment of correlated factors using other tools to assess psychological features, such as anxiety, depression, and self-esteem, that often coexist in children with overweight and obesity. A brief self-report measure was used for the assessment of the HRQoL dimensions; however, it may be interesting to use a mixed-methods approach to hear the children’s voices using other instruments. Finally, it may also be interesting to implement a repeated measures study with the opportunity to see how some of these measures might vary according to changes in individual BMI.

## Conclusion

Considering the worldwide increase in childhood obesity, sufficient enough to consider it a pandemic, our findings are relevant for educational research and practice. Teachers and educators are highly likely to encounter children with weight problems during their educational practice. Our results show that being overweight can impact children’s perceptions of school functioning and well-being. Children with overweight and obesity who show school dissatisfaction may also report dissatisfaction with themselves, their abilities, and their relationships with others, particularly in the contexts of family and friendships. However, because of the strong social network of teachers and peers, the school context is crucial in promoting the well-being of children, including academic well-being. Relationships with teachers and peers can bring a sense of safety and connectedness, ensuring that children with excess body weight do not “feel any different” from their peers. These relationships may positively impact children’s self-concept and, in turn, school and general well-being. Such improvements would be promoted by using evidence-based strategies: to help students improve their socioemotional well-being and connectedness with others, developing group activities focused on social and emotional competencies, creating opportunities for students to establish positive relationships, and understanding different perspectives. Furthermore, the school should consider implementing evidence-based educational activities that work at several levels to promote healthy eating, physical activity and healthy lifestyle, key components for reducing the risk of overweight and obesity, involving families and communities in promoting supportive healthy school and family environments (Piana et al., 2017). High parental involvement in school activities is associated with positive outcomes for children, such as academic satisfaction and higher levels of well-being (Suldo & Fefer, 2013).
